# Examining auditory modulations on detecting and pooling visual global motion

**DOI:** 10.3389/fpsyg.2025.1522618

**Published:** 2025-06-24

**Authors:** Yi-Chuan Chen, Ang-Ke Ku, Pi-Chun Huang

**Affiliations:** ^1^Department of Medicine, MacKay Medical College, New Taipei City, Taiwan; ^2^Department of Psychology, National Cheng Kung University, Tainan, Taiwan

**Keywords:** multisensory processing, audiovisual interactions, equivalent noise paradigm, internal noise, sampling efficiency, response bias

## Abstract

**Introduction:**

Multisensory signals often interact to reduce perceptual uncertainty in the environment. However, the effects and mechanisms underlying audiovisual interactions in motion perception remain unclear. In this study, we adopted the method of constant stimuli and the equivalent noise paradigm to investigate whether and how auditory motion influences the perception of visual global motion.

**Methods:**

The visual stimuli consisted of dots moving either up-left or up-right, with motion directions sampled from a normal distribution at five levels of standard deviation. The auditory stimuli were white noise moving either laterally (leftward or rightward; Experiment 1) or diagonally (up-left or up-right; Experiment 2), forming a coarse congruent or incongruent directional relationship with the visual motion trajectories. Stationary and no-sound conditions were also included. The auditory signals were task-irrelevant and presented in spatial proximity to, but not fully overlapping with, the visual stimuli. Participants had to discriminate the direction of the visual global motion.

**Results and discussion:**

After accounting for or eliminating the bias induced by auditory motion at the decisional level, the thresholds of visual motion perception were found to be similar across the four auditory conditions. Further analysis using the equivalent noise model confirmed that auditory motion did not influence the detection or pooling of visual motion signals. Hence, we did not find evidence to support the notion that auditory motion modulates the sensory or perceptual processing of visual global motion, delineating a boundary condition for such crossmodal interactions.

## 1 Introduction

Visual and auditory signals are not processed independently but often influence each other. Growing evidence suggests that the presentation of an auditory signal enhances the efficiency and/or accuracy of visual signal processing (Chen and Spence, 2011; Meredith and Stein, 1985; Miller, 1991; Van der Burg et al., 2008; Vroomen and de Gelder, 2000). These facilitatory effects plausibly arise from either the merging of multisensory signals at the subcortical or cortical levels during feedforward processing (Driver and Noesselt, 2008; Stein and Stanford, 2008), or through associative and/or inferential processes that integrate multisensory signals perceived as originating from the same source (Chen and Spence, 2017; Shams and Beierholm, 2010). In either case, audiovisual signals that coincide in space and time are more likely to integrate (Körding et al., 2007; Stein and Meredith, 1993). Given that motion signals combine both spatial and temporal features of an object or a group of objects, it is expected that auditory motion may also modulate visual motion perception. While vision often dominates in motion perception (e.g., Jain et al., 2008; Kitagawa and Ichihara, 2002), auditory influences can emerge when visual motion signals are weak or ambiguous (e.g., Alink et al., 2012; Chen et al., 2018; Kim et al., 2012). In the current study, we aimed to explore whether auditory motion signals modulate the early visual processing of global motion using a novel experimental paradigm designed to isolate sensory-level effects.

The random dot kinematogram (RDK) is commonly used to study visual global motion perception (Britten et al., 1992; Newsome et al., 1989; Newsome and Paré, 1988). In the RDK display, a certain proportion of dots are designated as signals, moving toward a specific direction, while the remaining dots act as noise, moving in random directions. Motion coherence thresholds are determined by the proportion of signal dots among the noise that the participant can detect or use to discriminate the direction of motion. That is, in the RDK, the perception is referred to as “global motion” because the global motion direction is not directly available from any single element but must be inferred by integrating motion signals across the stimulus array.

The RDK has also been used in previous studies to investigate the auditory modulation of visual motion perception. When a sound perceived as moving in a specific direction is presented alongside the RDK, it is generally assumed to influence the abstract representation of the visual global motion rather than the local motion of individual dots or objects. However, previous studies have reported conflicting results. For example, Meyer and Wuerger (2001) demonstrated that auditory motion did not influence the discrimination sensitivity of visual global motion but instead introduced a response bias toward the auditory direction. Specifically, improved visual performance when auditory and visual motions were congruent can be explained by the probability summation rule at the decisional level, rather than by the information integration at the perceptual level (see Alais and Burr, 2004a; Wuerger et al., 2003, for similar results in visual motion detection tasks). In contrast, Kim et al. (2012) designed a study where the direction of the sound was non-informative for a visual motion detection task, and the visual motion was always leftward. They demonstrated that accuracy was improved when a congruent sound was presented, compared to when an incongruent sound or no sound was provided. However, this facilitatory effect only occurred at a medium level of task difficulty (see also Chen et al., 2011a; Ross et al., 2007, for crossmodal facilitation at medium difficulty levels). Kim et al. (2012) suggested that a congruent sound enhanced the global motion signals at a mid-level coherence through a multiplicative effect at the sensory/perceptual level, as the non-informative sound was unlikely to induce any response bias. These contradictory findings in the RDK paradigm may be due to differences in how auditory motion relates to visual motion and the task design (e.g., congruent vs. incongruent, informative vs. non-informative, task-relevant vs. task-irrelevant), and the sound may influence different stages (e.g., sensory, perceptual, or decisional level) of visual motion processing. Hence, it is crucial to distinguish response bias from sensory/perceptual processing to prevent the former from overshadowing any effects of the latter.

**Figure 1:**
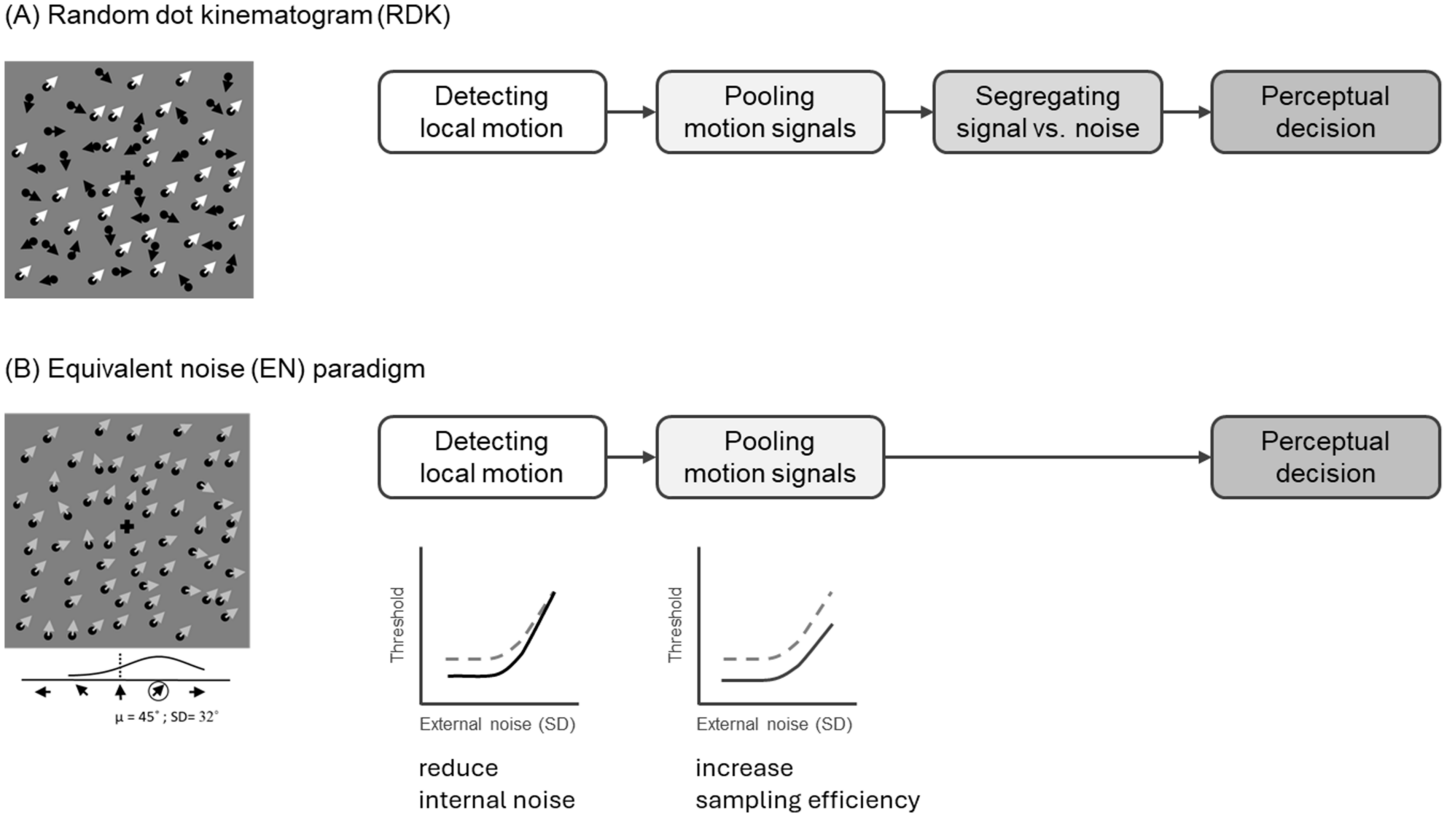
Schematic displays and internal processing of visual motion paradigms. **(A)** The random dot kinematogram (RDK) paradigm, where coherently moving dots (white arrows) represent the signal, while dots moving in random directions (black arrows) represent noise. This paradigm assumes four stages of processing to perceive visual global motion. **(B)** The equivalent noise (EN) paradigm involves dots moving in a mean direction (e.g., 45° in the figure) with varying levels of directional variability (e.g., a standard deviation of 32°). Facilitating local motion detection (lower-left panel) reduces internal noise, resulting in lower thresholds at lower levels of external noise (solid line), compared to when no such facilitation is present (dashed line). Conversely, facilitating the pooling of local motion (lower-right panel) enhances the overall sampling efficiency of the motion signal, thereby reducing thresholds across all levels of external noise (solid line), relative to the condition without such facilitation (dashed line). Notably, the EN paradigm bypasses the need for signal-noise segregation (Allard and Cavanagh, 2012; Dakin, 2001; Dakin et al., 2005).

Perceiving global motion in the RDK involves multiple stages of information processing (Figure 1A). First, local motion signals are detected in the primary visual cortex (V1), which is sensitive to the motion direction within a limited region (Hubel and Wiesel, 1968). Second, these local motion signals are pooled across space to estimate the direction of the global motion (Lund, 1988; Movshon and Newsome, 1996). The middle temporal (MT) visual area, or areas between V1 and MT, are involved in this pooling stage by integrating directionally tuned input from V1 (McCool and Britten, 2008). Third, for an observer to detect or discriminate global motion, motion signals must be segregated from, or exceed, the noise. Lastly, at the decisional level, when motion signals reach a certain threshold, a participant’s response bias may also influence judgments of perceived global motion.

In investigating the auditory modulation of visual global motion perception, we aimed to break down the sensory/perceptual mechanism into two components: detecting local motion and pooling motion signals. To achieve this, we adopted an alternative psychophysical method for studying global motion perception—the equivalent noise (EN) paradigm (also known as the voluntary averaging paradigm; Dakin, 1999, 2001; Dakin et al., 2005, 2009; Solomon, 2010). Originally developed to study orientation integration (Dakin, 1999), the EN paradigm has since been extended to global motion tasks (Dakin et al., 2005), allowing dissociation between internal uncertainty and pooling efficiency. In the EN paradigm, observers are required to discriminate the mean motion direction from an array sampled from a normal distribution with a specific variance of motion directions, without the presence of randomly moving dots. Given the multiple stages at which auditory motion could influence visual global motion perception in the RDK, the EN paradigm allows for a clearer distinction between detecting local motion signals and pooling these signals while bypassing the need to segregate coherent dots from random motion (Figure 1B).

Specifically, the EN paradigm is based on the variance summation model, which posits that both internal and external noise contribute to determining perceptual thresholds (see Allard and Cavanagh, 2012, for discussion). Internal noise refers to the random uncertainty inherent in the visual system (Barlow, 1978; Pelli, 1990); in the context of visual motion discrimination tasks, this uncertainty is related to the motion direction of individual dots (Dakin et al., 2005). External noise, on the other hand, refers to the variability in motion directions introduced in the task, specifically the standard deviation (SD) of the dot movements in the current study. Sampling efficiency reflects how effectively the visual system integrates local motion signals into a coherent global direction estimate. Higher efficiency indicates more precise integration across the motion field. When external noise is zero or lower than internal noise, thresholds are primarily determined by internal noise and sampling efficiency. Conversely, when external noise exceeds internal noise, performance is mainly driven by sampling efficiency. Hence, in the present study, a change in internal noise would indicate that auditory motion influences the detection threshold of local motion signals, while a change in sampling efficiency would suggest that auditory motion affects the ability to pool local motion signals. The EN paradigm therefore allows us to separate the sensory/perceptual parameters related to global motion processing into two components: internal noise and sampling efficiency.

To account for the auditory modulations at the decisional level, we estimated response bias using a two-alternative forced choice (2AFC) task with the constant stimuli method. This allowed us to chart a psychometric function describing the relationship between stimulus strength and response accuracy. In cases where participants could not discriminate the target, the guessing rate would approach 0.5; however, if auditory motion introduced a response bias, the guessing rate would differ between the congruent and incongruent conditions.

In sum, we used a visual motion discrimination task incorporating the constant stimuli method and the EN paradigm to investigate how auditory motion influences visual global motion perception in terms of response bias and threshold, with the latter further decomposed into internal noise and sampling efficiency. If auditory motion reduces internal noise in visual motion processing, lower thresholds are expected at lower levels of external noise (i.e., lower SD levels, as shown in the lower-left panel of Figure 1B). Conversely, if auditory motion enhances the sampling efficiency of visual motion signals, lower thresholds should be observed across all levels of external noise (i.e., all SD levels, shown in the lower-right panel of Figure 1B). In this framework, the influence of leftward or rightward auditory motion signals is hypothesized to occur during the feedforward stages of visual motion processing, prior to the formation of a global motion representation. This contrasts with the proposal by Spence and Chen (2012), which posits that crossmodal integration occurs only after unimodal grouping is complete. Our design aimed to test this distinction directly by using the EN paradigm to assess whether auditory motion modulates visual motion sensitivity at the level of internal noise or sampling efficiency. We compared four sound conditions: absent, stationary, congruent, and incongruent. The auditory stimuli were task-irrelevant and only informative by chance, allowing us to investigate the audiovisual interactions in a neutral state. The mean visual motion direction was tilted away from the upward direction (e.g., toward the up-left or up-right). In Experiment 1, the auditory motion signal moved horizontally—either from left to right or vice versa—to provide a strong leftward or rightward motion cue. In Experiment 2, in order to enhance the coherence between visual and auditory motion directions, the auditory motion signals were diagonal, moving from bottom-left to top-right or bottom-right to top-left. In both experiments, we estimated the parameters of visual global motion perception together with the guessing rate associated with response bias in the psychometric function, or with a fixed guessing rate of 0.5 for comparison.

## 2 Experiment 1

### 2.1 Methods

#### 2.1.1 Participants

Six observers (age range: 21–25 years old, one male), including one of the authors (AK), participated in Experiment 1. Written informed consent was obtained from all participants prior to the experiment. Participants were compensated for their participation and, except for the author, were unaware of the purpose of the experiment. All observers self-reported normal or corrected-to-normal vision and normal hearing. The study was carried out in accordance with the Declaration of Helsinki and was approve d by the National Cheng Kung University research ethics committee for human behavioral sciences (REC-HBS 104-135-2).

#### 2.1.2 Apparatus and stimuli

The experiment was conducted in a dimly lit room. Stimuli were presented using MATLAB (MathWorks) and Psychtoolbox-3 (Kleiner et al., 2013), via a Bits# Stimulus Processor (Cambridge Research System). Visual stimuli were displayed on a 19” CRT monitor (CXT VL951T) with a refresh rate set at 85 Hz, a resolution of 800 × 600 pixels, and a mean luminance of 31.92 cd/m^2^. The nonlinear output of the monitor was measured with a ColorCAL II Colorimeter (Cambridge Research System) and calibrated to ensure a linear response.

Auditory stimuli were presented through a pair of speakers (JBL JEMBE), delivered using an AudioFile Stimulus Processor (Cambridge Research System) to ensure synchronized onset of the auditory and visual stimuli. The monitor and speakers were 60 cm and 70 cm from the observers, respectively. The speakers were placed 10 cm to the left and right of the monitor, making them 56.5 cm apart from each other.

**Figure 2:**
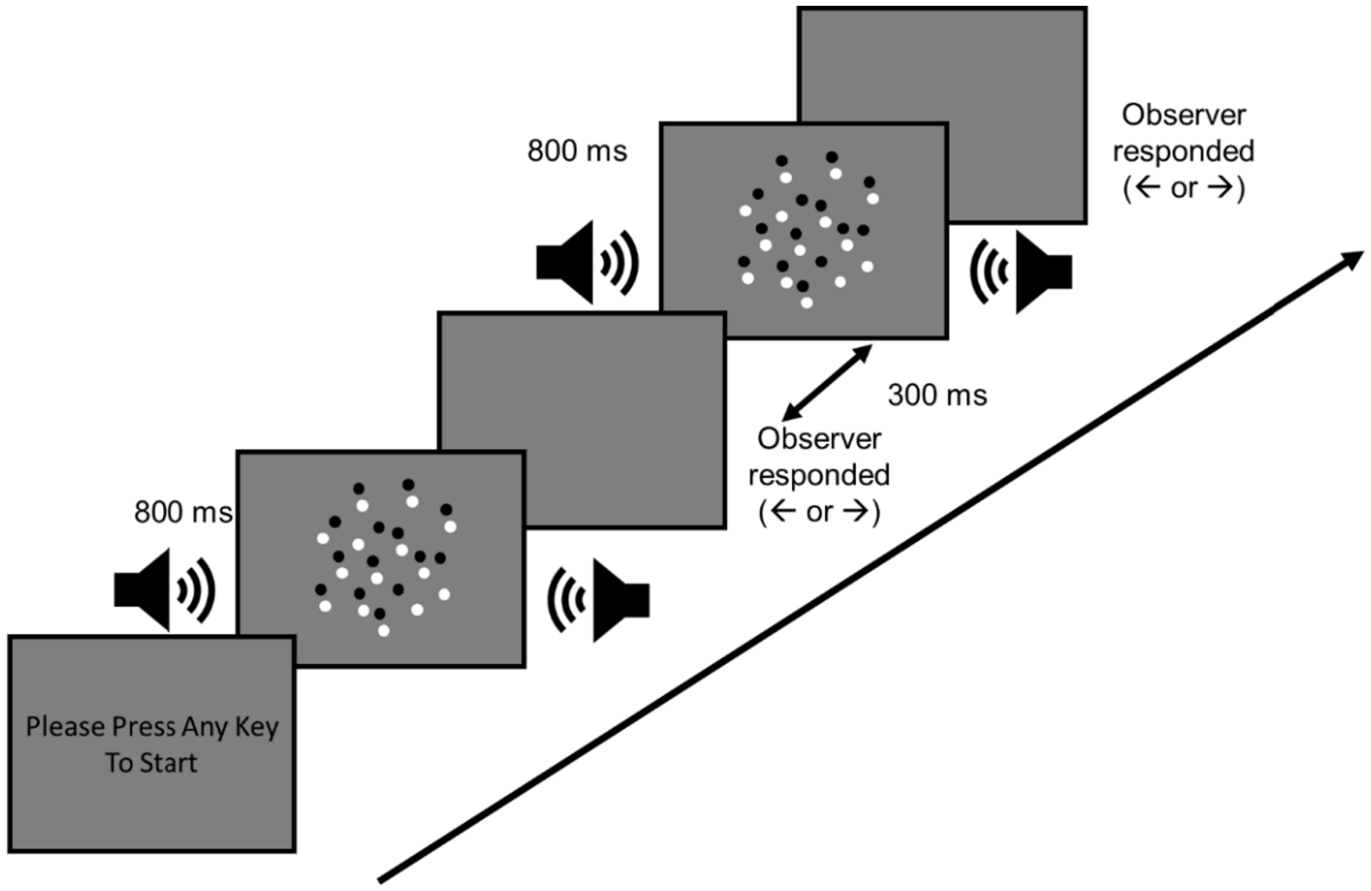
Schematic experimental procedure in Experiment 1. The visual and auditory stimuli displayed 800 ms synchronously. The visual dots moved toward either up-left or up-right, and the auditory motion was leftward or rightward. Observers responded by pressing a key during a blank frame. After 300 ms of response, the next trial started.

The visual stimuli comprised 100 dots with a diameter of 0.2° and distributed within an 8.4° visual angle for 800 ms (Figure 2). The dots included 50 bright and 50 dark dots with a Weber contrast value of ± 0.5, moving at a speed of 2°/s. To prevent the observers from tracking specific dots, each dot had a limited lifetime of 200 ms. Moreover, the lifetime and position of each dot in the first frame were randomized, causing dots to disappear in different frames. A central fixation point (a black cross) was always present during the experiment. The moving directions of the dots were sampled from a normal distribution with an SD of *σ* degrees and a mean direction of *μ* degrees, angled away from the upward motion direction. Five SD values (σ = 0°, 4°, 8°, 16°, and 32°) were tested, along with 10 mean directions selected from 14 possible levels (μ = ± 0.25°, ± 0.5°, ± 1°, ± 2°, ± 4°, ± 8°, and ± 16°), where positive values indicate the up-right direction and negative values indicate the up-left direction. For each SD level, the 10 mean directions were chosen based on individual performance, as participants’ thresholds varied.

The auditory stimulus consisted of an 800-ms burst of white noise, synchronized with the onset and offset of the visual motion. The volume of the white noise was 58.5 dB measured at the observer’s head position. The speed of the directional auditory stimuli was 54.94°/s, equivalent to 0.71 m/s. This speed was selected to produce a clearly perceivable motion trajectory while maintaining a comparable stimulus duration across modalities. A pilot experiment confirmed the effectiveness of this choice, with participants accurately identifying the direction of the auditory motion signal with approximately 98% accuracy. Audacity 2.0.6 was used to generate the auditory stimuli, either stationary or directional. Specifically, the white noise from two channels with equal amplitude sounds like static, and the amplitude in one channel faded in while the amplitude in the other channel faded out, creating a cross-fading effect that simulated movement toward the right or left.

The experimental design was based on the assumption that, for example, hearing a rightward-moving sound could enhance the perception of rightward visual motion, thereby aiding in the discrimination between up-right and up-left motion. In the congruent condition, up-right visual motion was paired with rightward auditory motion, while in the incongruent condition, up-right visual motion was paired with leftward auditory motion. Four sound conditions were tested in the experiment: absent, stationary, congruent, and incongruent. The first two served as control conditions, where the sound was either absent or provided no directional information related to the visual motion.

#### 2.1.3 Procedure

A 2AFC task with a constant stimuli method was used in this experiment. Both visual and auditory stimuli were displayed for 800 ms synchronously, after which the participants indicated the direction of the visual global motion by pressing a pre-designated key. The participants were instructed to attend to both visual and auditory stimuli but to respond only to the visual stimuli; that is, the auditory motion was task-irrelevant. They pressed the left or right arrow key to indicate whether the mean motion direction of the visual dots was toward up-left or up-right, respectively. The subsequent trial started 300 ms after the participant’s response.

Ten levels of mean direction, deviating from the vertically upward direction, were selected for each SD level. The SD was fixed in each run, and the four sound conditions were mixed on a trial-by-trial basis in a run. This approach ensured that the moving direction of the sound was only informative in a quarter of the trials. Each condition was presented 10 times within each run, giving rise to 400 trials (10 moving direction levels × 4 sound conditions × 10 trials). Three participants were tested across the five SD levels, increasing from 0° to 32°, while the other three were tested with SD at 0° and 32° in the first two runs, followed by increasing SD levels of 4°, 8°, and 16° in subsequent runs. They repeated each run for a particular SD four times. In total, there were 8,000 trials, which could have increased further if the data fit had been inadequate.

#### 2.1.4 Data analysis

##### 2.1.4.1 Psychometric function

**Figure 3:**
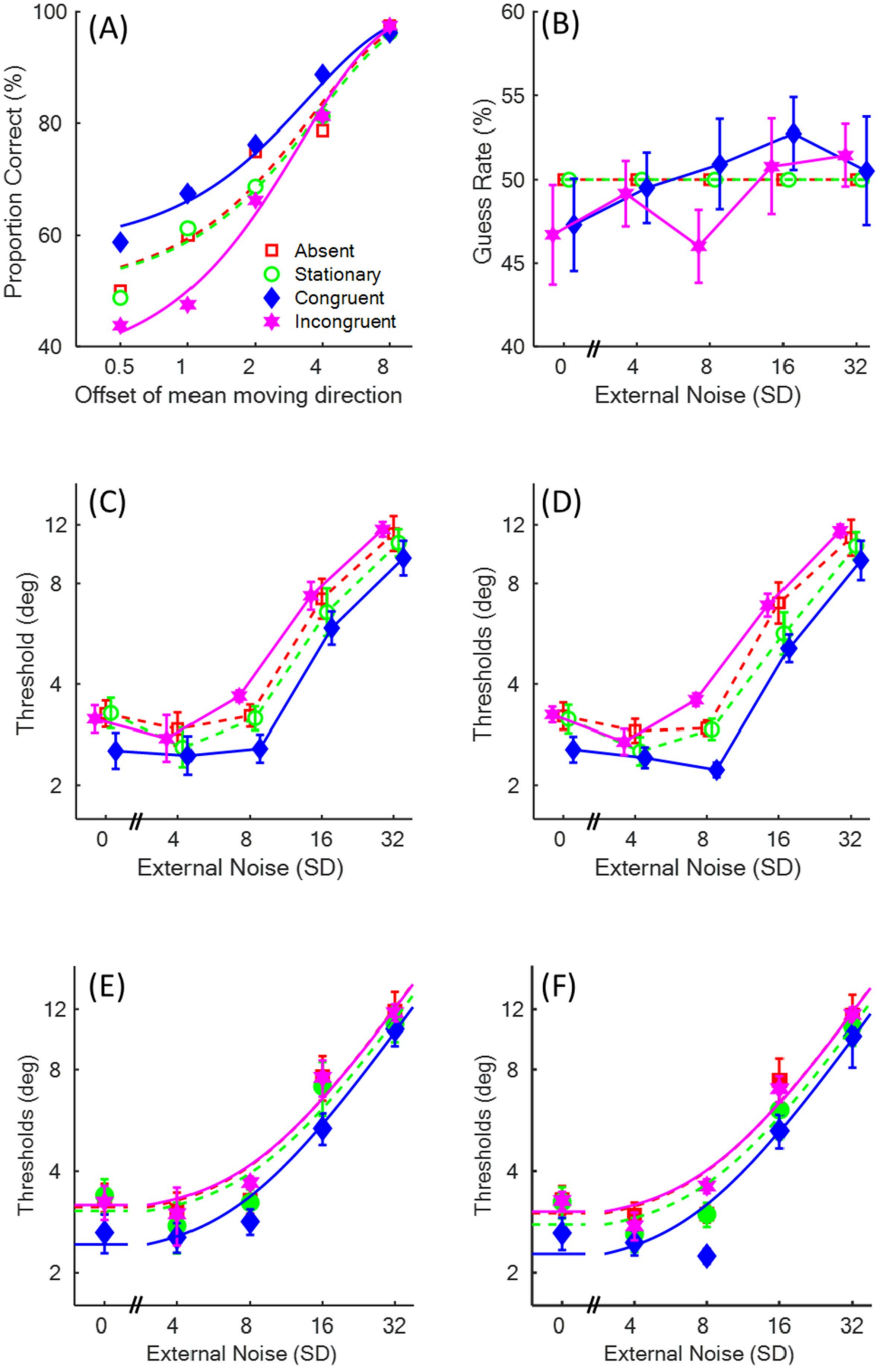
The results in Experiment 1. The red squares represent the absent condition, the green circles represent the stationary condition, the blue diamonds represent the congruent condition, and the magenta stars represent the incongruent condition. **(A)** An example of the psychometric function of one participant. **(B)** Mean guessing rates of 6 participants as a function of external noise (i.e., the standard deviation, SD) levels. **(C,E)** The mean Threshold vs. Noise (TvN) functions and the fitted curves of the equivalent noise (EN) model when the response bias was separately estimated in the congruent and incongruent conditions. **(D,F)** The TvN functions and the fitted curves of the EN model when the response bias was fixed at 0.5 in the four sound conditions.

The psychometric function was defined as the proportion of correct responses against the mean degrees deviating from the upward direction of the visual motion (see Figure 3A for an example). The accuracies of the up-left and up-right motions at the same degree of deviation from the vertical upward direction (i.e., the *μ*) were combined. In conventional data analysis of the EN paradigm, the psychometric function is defined as the proportion of perceived rightward motion against the offset degrees, ranging from left to right, of the vertical direction. In this context, the slope of the psychometric function is estimated as the threshold, while the point of subjective equality (PSE) is estimated as the response bias. That said, using this conventional analysis cannot estimate both thresholds and response biases across the four sound conditions in the same psychometric functions (see Supplementary material C).

In the current study, because we wanted to investigate the congruency effect between visual and auditory motions, we combined the responses for up-left and up-right visual motions at the same offset degrees and used the percentage of correct responses as the y-axis. Thus, the threshold values in our study were the deviation of the motion direction at which the participants achieved approximately 82% accuracy. Palamedes 1.8.1 (Prins and Kingdom, 2009) was used to estimate the psychometric function parameters for each participant. We used Equation 1:

(1) $$\begin{array}{l} \psi (x;\alpha ,\beta ,\gamma ,\lambda )=\gamma +(1-\gamma -\lambda )F(x;\alpha ,\beta )\\ =\gamma +(1-\gamma -\lambda )[1-\exp(-{\left(x/\alpha \right)}^{\beta })] \end{array}$$

where *F*(x; α, β) is the Weibull function; x is the stimulus intensity in the logarithmic unit, which is the offset degrees of vertical direction; α is the threshold of the function, indicating the degree of deviation that a participant was able to discern in the direction of motion; β is the slope of the function; γ is the guessing rate, and λ is the lapse rate. The psychometric functions for the four sound conditions (absent, stationary, congruent, and incongruent) at each SD level were fitted simultaneously, with the βs constrained to be equal across the four auditory conditions. In addition, the λs for each SD condition were held constant for each observer. In the congruent and incongruent conditions, γ was treated as a free parameter to estimate the response bias, while it was fixed at 0.5 for the absent and stationary conditions. The maximum likelihood method was used to derive the threshold and slope of the psychometric function. The bootstrapping method (N = 1,000) was used to calculate the standard deviation of the estimated parameters (α, β, γ, and λ) and assess the goodness-of-fit. The derived parameter α (i.e., the threshold) was used for the subsequent analysis.

##### 2.1.4.2 Equivalent noise model fitting

Each participant provided 20 threshold estimations (4 sound conditions × 5 SD levels), which were used to fit the equivalent noise (EN) model in order to estimate each participant’s internal noise (*σ_int_*) and sampling efficiency (*N_samp_*). The external noise (*σ_ext_*) was defined as the SD levels. Mean thresholds among the participants were also calculated to fit the EN model and are demonstrated in Figures 3E,F. The equation for the EN model can be written as follows Equation 2:

(2) $$\sigma_{\mathrm{obs}}=\sqrt{\frac{\sigma_{\mathrm{int}}^{2}+\sigma_{\mathrm{ext}}^{2}}{N_{\text{samp}}}}$$

The parameters, internal noise (*σ_int_*) and sampling efficiency (*N_samp_*), were estimated using least squares methods, which minimize the sum of squared errors (SSE) between the observed data and the predicted data. The equation for the SSE is as follows Equation 3:

(3) $$\mathrm{SSE}=\sum {\left[\log(\sigma_{\text{pred}})-\log(\sigma_{\mathrm{obs}})\right]}^{2}$$

where σ*_pred_* is the threshold predicted by the model, and σ_obs_ is the threshold derived from the psychometric function described above. We chose the parameters that resulted in the lowest SSE and converged to consistent values. We took the logarithm of the thresholds for two reasons. First, the standard errors of the thresholds at higher SD levels were greater than those at lower SD levels. To avoid an imbalance in weights, we employed the logarithmic transformation to linearize the thresholds. Second, the relationship between the physical stimulus (mean motion directions) and the psychological representation adheres to Weber-Fechner’s law. The fitting procedures were repeated 20 times with various initial parameter guesses. We fitted the results under the assumption that both internal noise (*σ_int_*) and sampling efficiency (*N_samp_*) varied across the four sound conditions. The averaged fitted parameters for Experiments 1 and 2 are reported in Table 1.

**Table 1:** Mean internal noise (𝜎_int_) and sampling efficiency (N_samp_) and one standard error (*SE*, in the parentheses) in Experiments 1 and 2.

Experiment	Response bias	Sound conditions			
		Absent	Stationary	Congruent	Incongruent
Internal noise (𝜎_int_)					
1	Estimated	8.78 (1.46)	9.70 (2.47)	9.83 (3.94)	8.38 (1.25)
	Fixed	8.78 (1.48)	9.02 (1.94)	8.19 (1.63)	8.09 (0.64)
2	Estimated	12.64 (1.23)	13.81 (1.21)	14.34 (1.65)	14.83 (1.36)
	Fixed	13.12 (1.10)	14.85 (1.47)	13.05 (1.40)	14.70 (1.29)
Sampling efficiency (*N_samp_*)					
1	Estimated	9.79 (2.13)	11.42 (2.56)	15.99 (5.39)	7.92 (0.85)	Fixed	10.50 (2.26)	11.94 (2.04)	15.00 (2.71)	8.26 (0.89)
	2	Estimated	30.91 (5.70)	33.84 (4.62)	43.41 (10.09)	38.49 (6.69)	Fixed	31.95 (5.36)	36.98 (4.63)	35.02 (7.47)	37.67 (6.07)

The response bias was 0.5 across the four sound conditions when they were fixed.

##### 2.1.4.3 Statistical analyses

The response biases (i.e., the estimated guessing rate, *γ*) were submitted to a two-way analysis of variance (ANOVA) on the factors of Congruency (congruent vs. incongruent) and SD level (five levels). Note that the guessing rates in the absent and stationary conditions were always fixed and not subject to analysis. Next, the estimated thresholds for visual motion discrimination were submitted to a two-way ANOVA on the factors of Sound (absent, stationary, congruent, and incongruent) and SD level (five levels). Finally, the estimated internal noise (*σ_int_*) and sampling efficiency (*N_samp_*) were each analyzed using a one-way ANOVA on the factors of Sound (absent, stationary, congruent, and incongruent). For all ANOVA tests, the Greenhouse–Geisser correction was applied when the assumption of sphericity was violated. Following any significant main effects or interactions, t-tests (two-tailed) with Bonferroni correction were conducted for *post-hoc* comparisons. Effect sizes were reported as partial eta squared (*η_p_^2^*) for ANOVAs and *Cohen’s d* for t-tests. In addition, Bayes factors (*BF_10_,* Morey, 2024) were calculated to quantify the evidence supporting the alternative hypothesis over the null hypothesis.

### 2.2 Results

We first examined whether auditory motion introduced a response bias in the visual motion discrimination task. If participants had no response bias, then the guessing rate (γ) at the most difficult condition (the 0.5° offset of mean moving direction in Figure 3A) should be close to 50%. If auditory motion induced a response bias, we would expect the guessing rate to be higher in the congruent than in the incongruent condition. Figure 3B demonstrates the mean guessing rate against the SD level. The estimated guessing rates in the congruent and incongruent conditions were submitted to a two-way repeated measure ANOVA on the factors of Congruency (congruent vs. incongruent) and SD level (five levels). The main effect of Congruency was significant [*F*(1,5) = 12.95, *p* = 0.016, *η_p_^2^* = 0.72, *BF_10_* = 0.35]; specifically, the guessing rate was significantly higher in the congruent (50.2%) than in the incongruent (48.8%) condition, though the Bayes Factor indicated that the evidence for this difference was weak. Neither the main effect of SD level [*F*(4,20) = 0.71, *p* = 0.596, *η_p_^2^* = 0.12, *BF_10_* = 0.23] nor the interaction of Sound and SD [*F*(1.85,9.25) = 0.52, *p* = 0.597, *η_p_^2^* = 0.09, *BF_10_* = 0.18] was significant. These results suggest that the congruency of auditory motion consistently biased participants’ judgments of visual motion direction (up-left or up-right direction, by pressing the auditory-motion compatible response keys) irrespective of the external noise level, indicating an auditory modulation at the decision level of information processing.

The participant’s mean thresholds across SD levels (Threshold vs. Noise function; TvN function) are plotted in Figure 3C, with the fitted curves of mean thresholds using the EN model demonstrated in Figure 3E (see Supplementary material A for individual participants’ TvN functions). The logarithmic threshold values were submitted to a two-way repeated measure ANOVA on the factors of Sound (absent, stationary, congruent, and incongruent) and SD level (five levels). The main effect of Sound [*F*(3,15) = 6.14, *p* = 0.006, *η_p_^2^* = 0.55, *BF_10_* = 0.11] reached significance; *post-hoc* comparisons with Bonferroni correction demonstrate a significant difference between congruent sound and stationary sound [*t*(5) = 6.56, *p* = 0.007, *Cohen’s d* = 2.69, *BF_10_* = 34.80], while only a marginal difference between congruent and incongruent sounds [*t*(5) = 3.94, *p* = 0.065, *Cohen’s d* = 1.61, *BF_10_* = 6.40]. The main effect of SD level was significant [*F*(1.54,7.70) = 38.38, *p* < 0.001, *η_p_^2^* = 0.89, *BF_10_* > 100]. *Post-hoc* pair-wise t-tests with Bonferroni correction revealed that the threshold was significantly higher in the 32° SD compared to 0°, 4°, and 8° SDs [*t*(5) > 6.75, *p*s ≤ 0.011, *Cohen’s d* > 2.75, *BF_10_* > 37.87], and higher in the 16° SD compared to 0° and 8° SDs [*t*(5) > 4.81, *p*s ≤ 0.048, *Cohen’s d* > 1.96, *BF_10_* > 12.07], demonstrating a typical trend for the TvN function. The interaction between Sound and SD level was not significant [*F*(12,60) = 0.66, *p* = 0.781, *η_p_^2^* = 0.12, *BF_10_* = 0.02]. These results suggest that the presentation of auditory motion improved sensitivity to visual motion perception when it was congruent with visual motion compared to a stationary sound.

Each participant’s internal noise and sampling efficiency were estimated using the EN model and then submitted to two separate one-way ANOVA on the factor of Sound (absent, stationary, congruent, and incongruent). The results showed that neither internal noises [*F*(1.21,6.04) = 0.31, *p* = 0.638, *η_p_^2^* = 0.06, *BF_10_* = 0.20] nor sampling efficiencies [*F*(1.30,6.51) = 2.02, *p* = 0.205, *η_p_^2^* = 0.29, *BF_10_* = 0.47] differed significantly across the four sound conditions.

In the design of Experiment 1, the four sound conditions were intermixed within a block of trials, meaning auditory motion was only congruent with visual motion in terms of direction in a quarter of the trials. Since the sound was task-irrelevant and only informative at the chance level, the response bias induced by the auditory motion should have been minimized. Thus, one might assume that the guessing rates across the four sound conditions would be the same and fixed at 0.5 (i.e., the guessing rate did not vary on a trial-by-trial basis). Based on this assumption, we reanalyzed the data with guessing rates fixed at 0.5 across all sound conditions in order to understand the consequences of equating response bias.

The logarithmic threshold values (Figure 3D) were submitted to a two-way repeated measure ANOVA on the factors of Sound and SD level. The most notable difference from the previous analysis, which included unequal response bias (Figure 3C), was that the main effect of Sound was significant [*F*(3,15) = 12.74, *p* < 0.001, *η_p_^2^* = 0.72, *BF_10_* = 0.17], and *post-hoc* pair-wise t-tests with Bonferroni correction revealed that the threshold was significantly lower in the congruent than in the rest of the sound conditions [*t*(5) > 4.96, *p*s ≤ 0.013, *Cohen’s d* > 2.02, *BF_10_* > 12.96]. Similar to the previous analysis that included unequal response biases, the main effect of SD level was significant [*F*(4,20) = 67.54, *p* < 0.001, *η_p_^2^* = 0.93, *BF_10_* > 100]. *Post-hoc* pair-wise t-tests with Bonferroni correction indicated that the threshold was significantly higher at the 32° SD compared to all smaller SD levels [*t*(5) > 9.24, *p*s ≤ 0.002, *Cohen’s d* ≥ 3.77, *BF_10_* > 100], and higher at the 16° SD than all smaller SD levels [*t*(5) ≥ 5.89, *p*s ≤ 0.020, *Cohen’s d* > 2.40, *BF_10_* > 23.47]. The interaction between Sound and SD level remained insignificant [*F*(12,60) = 1.86, *p* = 0.058, *η_p_^2^* = 0.27, *BF_10_* = 0.03].

Even more critically, the smaller threshold in the congruent condition, after being fitted with the TvN function, was attributable to a significant difference in sampling efficiencies [see Table 1, *F*(3,15) = 4.81, *p* = 0.015, *η_p_^2^* = 0.49, *BF_10_* = 0.76]. However, *post-hoc* pair-wise t-tests with Bonferroni correction showed no significant difference among the four sound conditions [*t*(5) < 2.93, *p*s ≥ 0.197, *Cohen’s d* < 1.19, *BF_10_* < 2.85]. Internal noise, on the other hand, was similar across the four sound conditions [*F*(3,15) = 0.31, *p* = 0.817, *η_p_^2^* = 0.06, *BF_10_* = 0.20].

Hence, if the response bias parameters in the congruent and incongruent conditions had not been estimated separately, their influence would have exaggerated the difference in estimated thresholds between the two conditions. This, in turn, may have inflated the differences in sampling efficiency across the four sound conditions and falsely suggested auditory modulation of the pooling of visual motion signals, when the effect actually stemmed from uncorrected response bias.

Notably, when response biases in the congruent and incongruent conditions were considered, the statistical results of their difference appeared equivocal: the *p*-value from the t-test exceeded the criterion (0.05) after correction for multiple comparisons, whereas the effect size and the Bayes factor indicated moderate evidence for an auditory modulatory effect. We believe the lack of statistical significance may be due not only to the small sample size but also to the incongruence of motion direction across modalities. Therefore, rather than solely increasing the number of participants, we opted for a more effective approach by enhancing the coherence between visual and auditory motion cues, which motivated the design of Experiment 2.

## 3 Experiment 2

In Experiment 2, two essential modifications were made to the experimental design: First, coherence between visual and auditory motion directions was enhanced by presenting both types of motion in similar directions, either up-left or up-right. To generate corresponding auditory motions, two pairs of speakers—each controlled by independent channels—were positioned diagonally (see below). Second, the sample size was enlarged. Based on the t-test of thresholds between the congruent and incongruent conditions in Experiment 1 (*Cohen’s d* = 1.58, *α* was set at 0.05, and *β* was set at *0*.95), the estimated sample size required to reach significance is over eight participants (calculated using G-Power 3.1, Faul et al., 2007). As a result, we doubled the number of participants compared to Experiment 1 to ensure sufficient statistical power.

### 3.1 Methods

#### 3.1.1 Participants, apparatus, and stimuli

Twelve observers (age range: 20–25 years old, 6 male) participated in Experiment 2. The visual stimuli were displayed on a 24.5” LCD monitor (Acer XB253Q GP) with a refresh rate set at 60 Hz, a resolution of 1920 × 1,080 pixels, and a mean luminance of 117 cd/m^2^. Sounds were delivered independently through four channels using a CREATIVE Sound Blaster X3 and two pairs of speakers (EDIFIER G2000). The monitor and speakers were 75 cm and 85 cm from the observers, respectively. The speakers were arranged in the four corners around the monitor, spaced 39 cm apart horizontally, 52 cm vertically, and 65 cm diagonally between each pair.

Compared to Experiment 1, the visual motion signal consisted of 300 denser, more uniform Gaussian blobs (approximating a difference-of-Gaussians profile with standard deviations of 0.025° and 0.05°) distributed within a 10° visual angle. The blobs, with a contrast value of 0.5, moved at a speed of 3°/s. The duration of the visual display was shortened to 500 ms, which was expected to limit the availability of visual motion information and thereby increase the likelihood that sound would influence visual performance. A black oval was presented in the center of the monitor before the trial started. The moving directions of the dots were sampled from five SD levels (*σ* = 0°, 4°, 8°, 16°, and 32°) and 11 mean directions selected from 15 levels (i.e., *μ* = 0°, ± 0.25°, ± 0.5°, ± 1°, ± 2°, ± 4°, ± 8°, and ± 16°), where positive values represented the up-right direction and negative values the up-left direction. Data from μ = 0° were excluded from analyses.

To synchronize with the onset and offset of the visual motion, the auditory stimulus was also a white noise presented for 500 ms. Notably, this 500 ms duration exceeds the minimal 200 ms required for the Minimal Audible Movement Angle (MAMA) to reach its maximum sensitivity (1.5°; Carlile and Leung, 2016). The volume was 65.7 dB measured at the participants’ head position. The speed of the directional auditory stimuli was 98.24^°^/s (equivalent to 0.87 m/s), faster than in Experiment 1. The white noise was edited with cross-fading effects across each pair of channels to create a rightward or leftward moving sound. The most critical difference from Experiment 1 was the auditory motion directions: each pair of speakers was positioned diagonally (bottom-left to top-right, and bottom-right to top-left), creating auditory motion either up-right or up-left at a 30° angle from the center of the monitor (calculated based on the speaker locations; see above). All other details were consistent with Experiment 1.

#### 3.1.2 Design and procedure

Four sound conditions were tested in this experiment: absent, stationary, congruent, and incongruent. In the congruent condition, the sound and the visual dots moved in a similar direction (e.g., both moving up-right). In the incongruent condition, the sound and the visual dots moved in orthogonal directions (e.g., the sound moved up-right while the visual dots moved up-left). In the stationary condition, the white noise was delivered from four speakers at equal volume. The amplitudes of the sounds were equalized across the congruent, incongruent, and stationary conditions. In the absent condition, no sound was presented with the visual motion array.

The experimental procedure was identical to that of Experiment 1, with the order of SD levels randomized across participants. Before the main experiment, we confirmed that each participant could correctly identify the direction of the auditory motion (up-left, up-right, or stationary) with over 80% accuracy. Data analysis followed the same procedure as in Experiment 1.

### 3.2 Results

**Figure 4:**
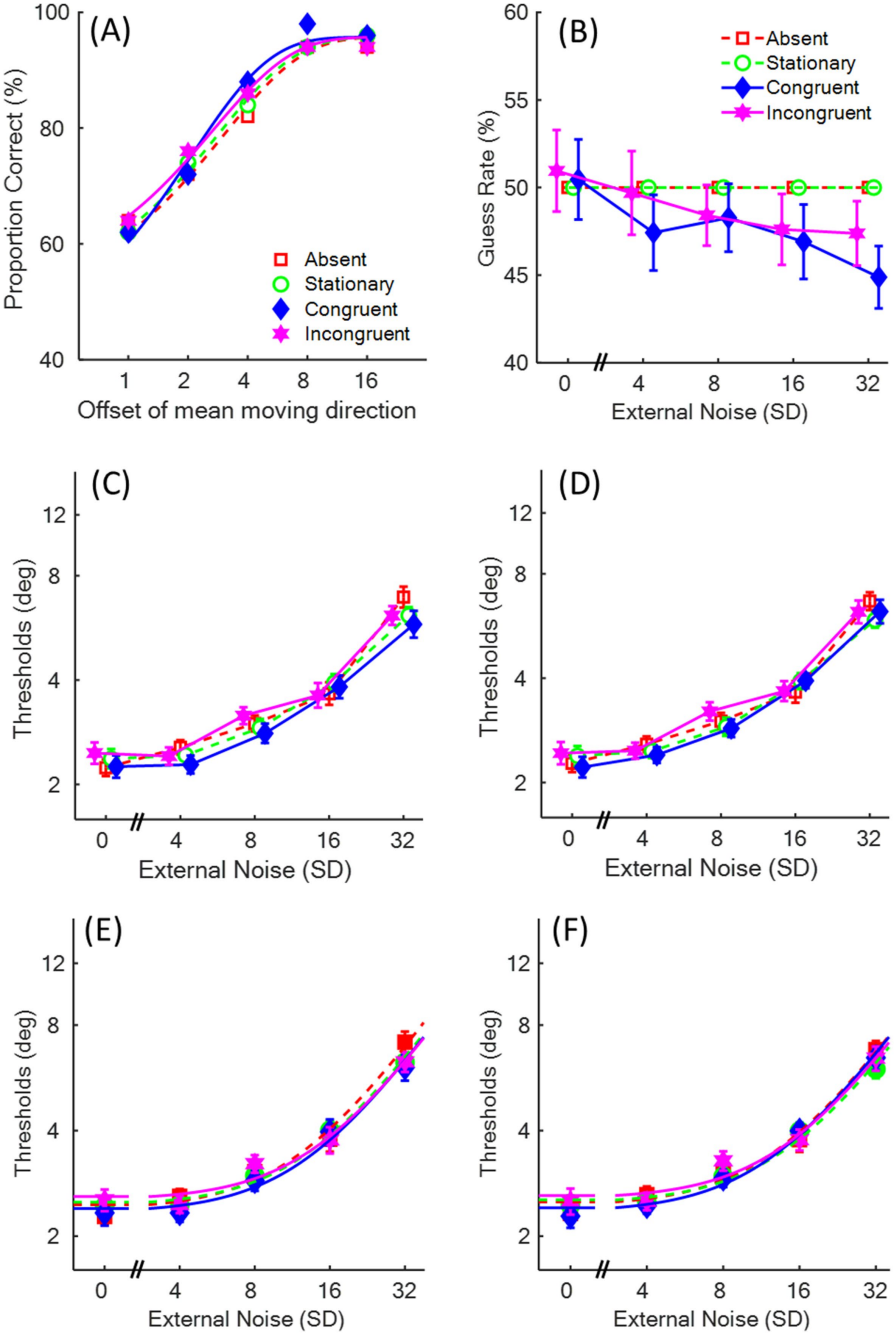
The results in Experiment 2. The red squares represent the absent condition, the green circles represent the stationary condition, the blue diamonds represent the congruent condition, and the magenta stars represent the incongruent condition. **(A)** An example of the psychometric function of one participant. **(B)** Mean guessing rates of 12 participants as a function of external noise (i.e., the standard deviation, SD) levels. **(C,E)** The mean Threshold vs. Noise (TvN) functions and the fitted curves of the equivalent noise (EN) model when the response bias was separately estimated in the congruent and incongruent conditions. **(D,F)** The TvN functions and the fitted curves of the EN model when the response bias was fixed at 0.5 in the four sound conditions.

Figure 4A illustrates a participant’s psychometric functions for the four sound conditions, showing the proportion of correct responses as a function of the mean degrees deviating from the upward direction of the visual motion. In the first analysis, guessing rates were estimated separately for the congruent and incongruent conditions, while those in the absent and stationary conditions were fixed at 0.5. The guessing rates in the congruent and incongruent conditions were submitted to a two-way repeated measure ANOVA on Congruency (congruent vs. incongruent) and SD level (five levels; see Figure 4B). Neither the main effects of Congruency [*F*(1,11) = 2.58, *p* = 0.136, *η_p_^2^* = 0.19, *BF_10_* = 0.28] nor SD level [*F*(4,44) = 0.74, *p* = 0.573, *η_p_^2^* = 0.06, *BF_10_* = 0.18] reached significance. Their interaction was also insignificant [*F*(4,44) = 1.00, *p* = 0.418, *η_p_^2^* = 0.08, *BF_10_* = 0.08]. These results indicate that, unlike in Experiment 1, the presentation of the sound moving either up-left or up-right did not elicit a response bias in judging the visual motion direction. This lack of bias is plausible because the auditory motion direction was highly congruent with the visual motion and/or incompatible with the response format (i.e., pressing either the left or right arrow key).

The logarithmic thresholds across participants (Figure 4C, see Supplementary material B for individual participants’ TvN functions) were submitted to a two-way repeated measure ANOVA on the factors of Sound (absent, stationary, congruent, and incongruent) and SD level (five levels). The main effect of Sound was not significant [*F*(3,33) = 1.10, *p* = 0.362, *η_p_^2^* = 0.09, *BF_10_* = 0.03], and the Bayes factor provided strong evidence in favor of the absence of sound effect. The main effect of SD level was significant [*F*(4,44) = 85.97, *p* < 0.001, *η_p_^2^* = 0.89, *BF_10_* > 100]. *Post-hoc* pair-wise t-tests with Bonferroni correction demonstrated that thresholds were significantly higher for the 32°, 16°, and 8°-SD levels compared to their respective lower SD levels [all *t*(11) ≥ 4.13, *p*s ≤ 0.017, *Cohen’s d* > 1.19, *BF_10_* > 26.26]. The interaction between Sound and SD level was not significant [*F*(12,132) = 1.53, *p* = 0.120, *η_p_^2^* = 0.12, *BF_10_* = 0.008].

Finally, thresholds at each SD level in the four sound conditions were fitted with the EN models (Figure 4E). The estimated internal noise and sampling efficiency (Table 1) were separately submitted to a one-way ANOVA on the factor of Sound (absent, stationary, congruent, and incongruent). Results showed that neither internal noise [*F*(3,33) = 1.24, *p* = 0.311, *η_p_^2^* = 0.10, *BF_10_* = 0.17] nor sampling efficiency [*F*(3,33) = 1.18, *p* = 0.333, *η_p_^2^* = 0.10, *BF_10_* = 0.19] reached significance.

Given the absence of significant response bias between congruent and incongruent conditions in Experiment 2, we fit the psychometric function again with a fixed guessing rate of 0.5 across all four sound conditions to derive the thresholds. Figures 4D,F show the thresholds and the fitted TvN functions of the EN model, respectively. The new logarithmic thresholds were then submitted to a two-way repeated measure ANOVA on the factors of Sound and SD level. The results remained consistent: a significant main effect of SD level [*F*(4,44) = 133.36, *p* < 0.001, *η_p_^2^* = 0.92, *BF_10_* > 100]. *Post-hoc* pair-wise t-tests with Bonferroni correction demonstrated that thresholds were significantly higher for the 32°, 16°, and 8°-SD levels compared to their respective lower SD levels [all *t*(11) > 4.59, *p*s ≤ 0.008, *Cohen’s d* > 1.32, *BF_10_* > 50.15]. There was no significant main effect of Sound [*F*(3,33) = 0.44, *p* = 0.729, *η_p_^2^* = 0.04, *BF_10_* = 0.02] or their interaction [*F*(12,132) = 1.67, *p* = 0.082, *η_p_^2^* = 0.13, *BF_10_* = 0.008]. Additionally, neither internal noise [*F*(3,33) = 1.27, *p* = 0.302, *η_p_^2^* = 0.10, *BF_10_* = 0.18] nor sampling efficiency [*F*(3,33) = 0.37, *p* = 0.774, *η_p_^2^* = 0.03, *BF_10_* = 0.13] significantly differed across the sound conditions.

## 4 General discussion

We investigated the potential mechanisms underlying auditory modulation of visual global motion perception using the constant stimuli method and the EN paradigm. Thresholds and response biases from the discrimination task of the visual global motion (up-left or up-right) were compared across four sound conditions (absent, stationary, congruent and incongruent). We then applied the EN model to assess whether internal noise and/or sampling efficiency varied with the sound manipulations. When the auditory motion was directed left or right, it induced response biases at the decisional level based on congruency. Logarithmic thresholds were similar at low SD levels but increased at higher SD levels, forming a typical TvN function; however, auditory motion appeared to have no significant effect on the threshold, internal noise, or sampling efficiency of visual motion perception (Experiment 1). When the auditory motion was designed to be more congruent with the visual motion (i.e., both moving up-left and up-right) than in Experiment 1, the induced response bias was eliminated, yet no significant auditory modulation on the threshold, internal noise, or sampling efficiency of visual motion perception remained. Taken together, we found no evidence supporting an interaction between visual and auditory motion at the sensory/perceptual level in terms of motion direction discrimination (Alais and Burr, 2004a; Meyer and Wuerger, 2001; Wuerger et al., 2003).

The results from estimating thresholds and response biases in the psychometric functions demonstrate that task-irrelevant auditory motion, which was only informative at the chance level, did not influence the threshold of visual global motion perception. Instead, auditory motion in the horizontal direction (left or right) induced a response bias, with participants more likely to select the correct answer in the congruent than in the incongruent condition. This suggested that the participants tended to report the auditory motion direction when uncertain about the direction of visual global motion. This is consistent with the auditory modulation of visual global motion detection/discrimination at the decisional level (Alais and Burr, 2004a; Meyer and Wuerger, 2001; Wuerger et al., 2003). However, this response bias was eliminated when the auditory motion direction (up-left or up-right) did not align with the response type (left-or right-arrow key).

The EN paradigm enabled us to investigate whether auditory motion modulates internal noise or sampling efficiency of visual global motion perception. Internal noise can be considered as the uncertainty in local motion signals during global motion discrimination (Dakin et al., 2005), while sampling efficiency reflects how effectively the visual system integrates these local signals into a global motion estimate (Dakin et al., 2005; Manning et al., 2014, 2015; Tibber et al., 2015). The human visual system is generally an inefficient sampler (Pardhan et al., 1996; Simpson et al., 2003), with higher sampling efficiency linked to better integrating local signals into a coherent global perception. Notably, the spurious auditory effect on sampling efficiency observed in Experiment 1 was eliminated after accounting for response bias, suggesting that this effect, when observed, may stem from decisional rather than sensory-level processes.

That said, our results using the EN paradigm differ from findings in studies employing the RDK to demonstrate auditory modulation effects on the sensory/perceptual processing of visual global motion. In Kim et al. (2012), auditory motion enhanced the accuracy of global motion detection, and in Hidaka et al. (2011), auditory motion canceled out the visual global motion in the opposite direction. Unlike the EN paradigm, RDK requires segregating the signal (coherently moving dots) from noise (randomly moving dots, see Figure 1A). This distinction suggests that auditory motion may facilitate the signal-noise segregation process in the congruent direction or inhibit it in the incongruent direction, a processing stage not captured by the EN paradigm.

Auditory motion modulations have also been reported in other visual motion paradigms. For example, a directional auditory signal can induce a static visual object to appear in motion, especially in the peripheral visual field (Teramoto et al., 2012; see a similar effect with the adaptation paradigm in Teramoto et al., 2010). In Teramoto et al.’s studies, the auditory signals modulated the local motion of a visual object, contrasting with our current results that local motion (indexed by the internal noise) was insensitive to the auditory motion. This difference can be explained by dissociating *position coding* and *motion direction coding*, which underpin motion perception. In the single-object paradigm (Teramoto et al., 2010, 2012), both types of coding contributed to visual apparent motion perception. In contrast, in the multiple-dot display used in the current study, the position coding was minimized due to all of the dots moving continuously. Thus, Teramoto et al.’s results likely reflect auditory influence on position coding, where sound alters the perceived position of the object, thereby modulating apparent motion.

In other studies, auditory motion direction modulated the perception of bistable visual motions, such as bidirectional visual apparent motion and the dominance in binocular rivalry with dichoptic contrasting motions (Alink et al., 2012; Conrad et al., 2010). In these studies, auditory motion likely influenced perception by resolving ambiguity in a top-down manner (e.g., through attention or association), rather than enhancing visual motion detection or integration during feed-forward processing (Chen et al., 2011b; Van Ee et al., 2009). This conjecture is consistent with neuropsychological evidence demonstrating that audiovisual interactions in motion perception can occur in the higher-order multisensory associated areas, such as the superior temporal gyrus and the supra-marginal gyrus (Baumann and Greenlee, 2007), subsequently amplifying processing in modality-specific areas (Gleiss and Kayser, 2014; Kayser et al., 2017; see Sadaghiani et al., 2009 for the dissociation of the bottom-up and top-down processing of audiovisual motion perception).

The EN paradigm is based on the variance summation model, which assumes a consistent pooling process across different external noise levels. However, Allard and Cavanagh (2011, 2012) demonstrated that this assumption may not hold true for tasks like luminance detection and mean orientation discrimination when using the EN paradigm. While it remains uncertain whether our audiovisual global motion task violated the noise-invariant assumption, it is unlikely to influence our conclusion: Auditory motion appeared not to influence internal noise or sampling efficiency in visual global motion perception, as the TvN functions remained stable across the four sound conditions.

We suggest that whether auditory motion signals can modulate visual global motion in the EN paradigm remains inconclusive, partly due to several discrepancies between visual and auditory motion stimuli that may have reduced the likelihood of their interaction. First, the motion directions across modalities were misaligned. The auditory motion provided leftward or rightward signals at ±90° in Experiment 1 and ±30° in Experiment 2, while the visual motion direction varied trial-by-trial between 0° and ±16°. Second, the visual and auditory motions were spatially disparate. Due to space limitations for the experimental setup, the speakers were placed outside and behind the monitor, causing the auditory motion to start and end at more peripheral and distant locations compared to the visual motion. Although a spatial ventriloquism effect—where a visual stimulus captures the perceived location of an auditory stimulus—may have occurred (Gardner, 1968; Hendrickx et al., 2015; Jackson, 1953), we did not assess participants’ perceived locations of the auditory motion stimuli, leaving this possibility unexamined. Third, the motion speed estimated from the experimental setup and stimulus presentation differed between modalities, with the auditory motion generally being faster than the visual motion. As interactions between visual and auditory motion speeds have been rarely studied and are difficult to predict given the changes across both spatial and auditory domains, this mismatch introduces additional uncertainty. Taken together, it is plausible that enhancing coherence between visual and auditory motions in terms of direction, location, and speed would strengthen their interactions during feedforward processing, as we aimed to test in the current study. Furthermore, high audiovisual coherence may also promote common-source assumptions, leading to a unified audiovisual motion representation (e.g., Chen and Spence, 2017; Shams and Beierholm, 2010). Increasing motion coherence, as well as requiring participants to attend and respond to both auditory and visual signals, could facilitate their interaction/integration (e.g., Wuerger et al., 2003). Importantly, the outcomes of audiovisual integration may not only manifest as improved detection thresholds but also as enhanced perceptual precision, reflected by reduced variability (Alais and Burr, 2004b). Future studies should aim to minimize discrepancies in motion direction, location, and speed between modalities, increase the task relevance of auditory and visual motion signals, and measure both the accuracy and variability of participants’ perceptual judgments.

In conclusion, our study did not provide evidence that auditory motion modulates the sensory/perceptual stages of visual global motion processing, specifically in terms of internal noise and sampling efficiency. These findings highlight the importance of accounting for response biases when using threshold-based models like the EN paradigm. Future work should seek to maximize crossmodal coherence and task relevance to assess audiovisual motion integration in terms of accuracy and precision performance.

## Data Availability

The raw data supporting the conclusions of this article will be made available by the authors, without undue reservation.
