# Supplementary material for: Examining auditory modulations on detecting and pooling visual global motion
Source: Front Psychol. 2025 Jun 24;16:1522618. doi: 10.3389/fpsyg.2025.1522618 (PMC12234566; doi:10.3389/fpsyg.2025.1522618)
Supplement: Supplementary file 1 [file Data_Sheet_1.pdf]

frontiers

Examining Auditory Modulations on Detecting and Pooling Visual Global Motion

Yi-Chuan Chen[1], **Ang-Ke Ku**[2], **Pi-Chun Huang**[2]

[1]Department of Medicine, MacKay Medical College, New Taipei City, Taiwan

[2]Department of Psychology, National Cheng Kung University, Tainan, Taiwan

*** Correspondence:**
Pi-Chun Huang
pichun_huang @mail.ncku.edu.tw

To complement the group-level results presented in the main text, we provide individual Threshold vs. Noise (TvN) functions for each participant in Experiment 1 (A) and Experiment 2 (B). In addition, an alternative analytical approach is presented in (C) to further examine the effects of auditory motion across both experiments.

A. Experiment 1: Individual TvN functions and EN model fits

Figure S1 shows the TvN functions for each participant in Experiment 1 under four sound conditions (absent, stationary, congruent, and incongruent). The fitted curves from the Equivalent Noise (EN) model are overlaid on the empirical data. These plots correspond to the average results presented in Figure 3 of the main text and illustrate individual variations in threshold patterns and model fits.

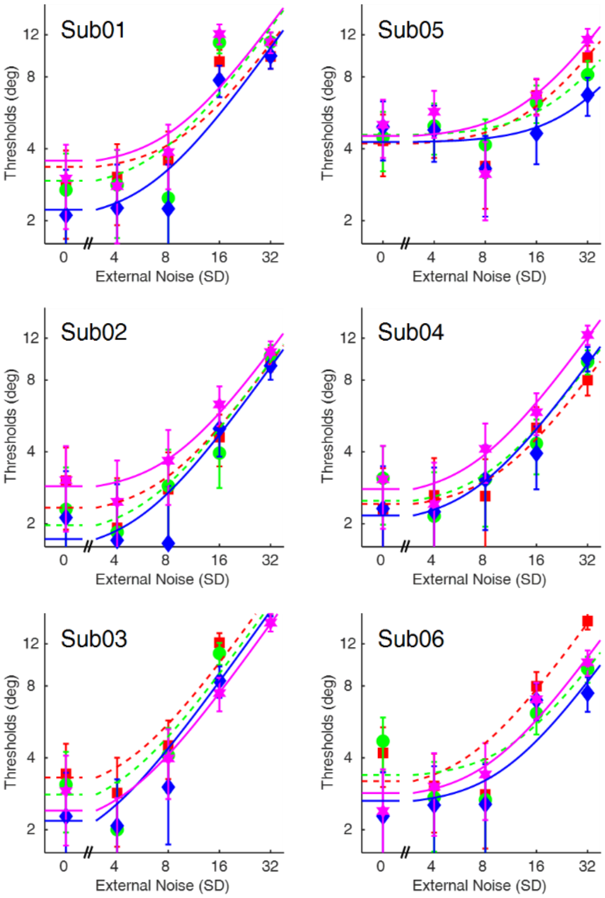

Figure S1. Individual threshold-versus-noise (TvN) functions and Equivalent Noise (EN) model fits for Experiment 1. Each panel shows data from one participant (Sub01–Sub06), plotting motion discrimination thresholds (in degrees) as a function of external noise (standard deviation, SD) across four sound conditions: absent (red square, dashed line), stationary (green circle, dashed line), congruent (blue diamond, solid line), and incongruent (magenta star, solid line). Error bars represent standard errors across repetitions. Curves reflect the best-fitting TvN functions derived from the EN model for each condition.

B. Experiment 2: Individual TvN functions and EN model fits

Figure S2 presents the same analyses as in (A), but for Experiment 2. These plots correspond to the group-level data shown in Figure 4 and again confirm that the auditory motion did not systematically affect individual participants' thresholds or EN model parameters.

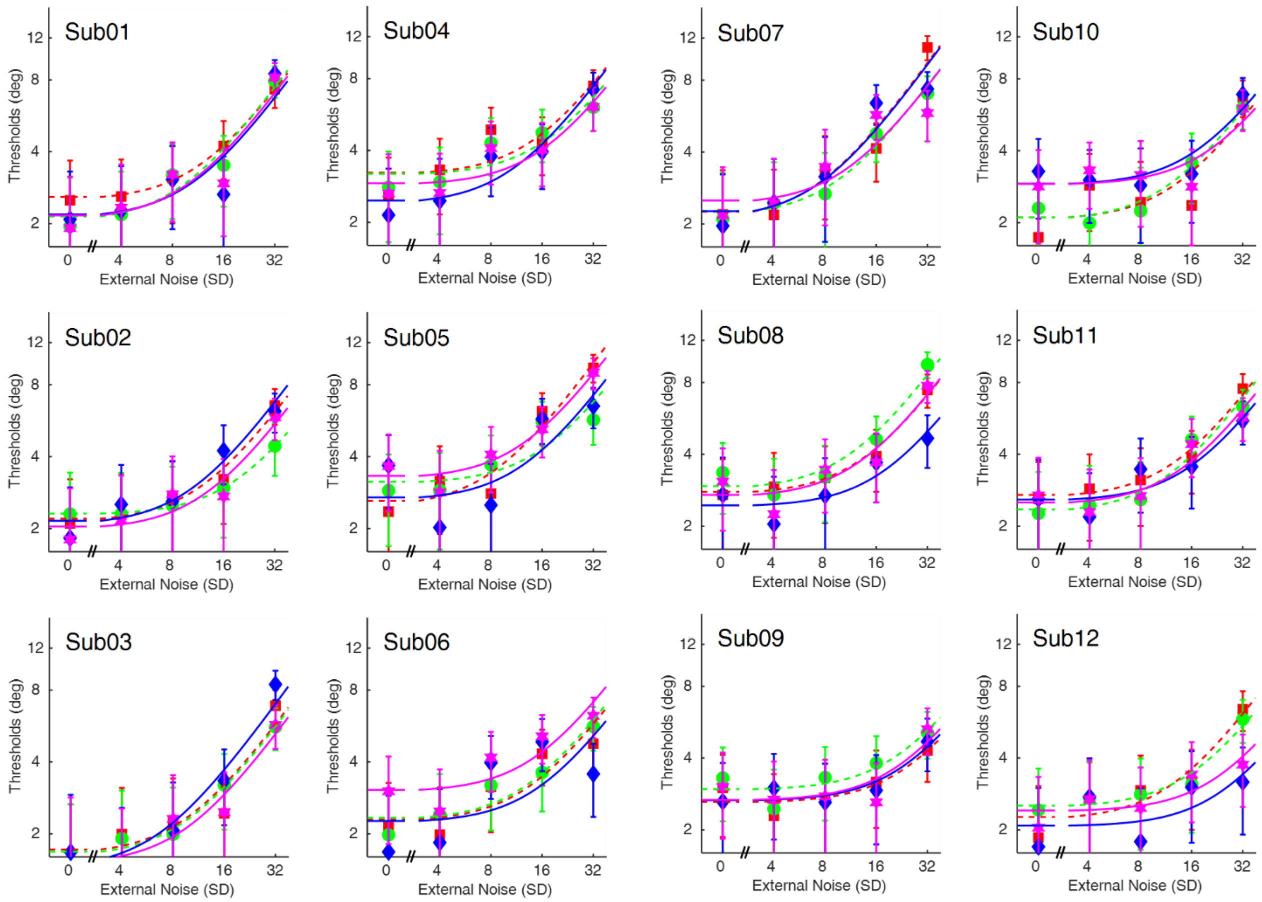

Figure S2. Individual threshold-versus-noise (TvN) functions and Equivalent Noise (EN) model fits for Experiment 2. The layout and plotting conventions are identical to those in Figure S1, with each panel showing one participant's data. Thresholds are plotted as a function of external noise (SD) across four sound conditions: absent (red square, dashed line), stationary (green circle, dashed line), congruent (blue diamond, solid line), and incongruent (magenta star, solid line).

C. Alternative data analysis approach across both experiments

We employed conventional methods to define the psychometric function as the proportion of perceived rightward motion relative to the actual motion direction from leftward to rightward. To further investigate the influence of auditory motion, we conducted two sets of analyses. The first focused on sound moving either right or left, which examined the point of subjective equality (PSE) that represents how auditory motion direction (left or right) influences response bias. The second analysis compared congruent and incongruent directions between auditory and visual motions, focusing on the slope of the psychometric function that indicates the threshold at which participants can reliably discriminate the direction of visual global motion.

Palamedes 1.8.1 (Prins & Kingdom, 2009) was used to estimate the parameters of psychometric functions for each participant. The psychometric function was modelled using the following equation:

$$\psi(x; \alpha, \beta, \gamma, \lambda) = \gamma + (1 - \gamma - \lambda)F(x; \alpha, \beta) \qquad (1)$$

where $F(x; \alpha, \beta)$ represents the cumulative normal distribution function; x denotes motion direction; α is the PSE, and β defines the slope of the function; γ and λ are the lower and higher bounds of the function, respectively. Psychometric functions across the four auditory conditions with the same SD level were fitted simultaneously, with γ and λ held constant and other parameters set as free. The value of β is the reciprocal of the standard deviation of the cumulative normal distribution. The maximum likelihood method was used to derive the α and β of the psychometric function. To assess parameter reliability, the bootstrapping method (N = 1000) was used to calculate the standard deviation of the estimated parameters (α, β, γ, and λ) and the goodness-of-fit. The derived parameter α (PSE) and the reciprocal of the β (1/slope) were used for the subsequent analyses. An example of a psychometric function for one participant from Experiment 2 is shown in Figures S3A and S3B, illustrating auditory motion directions of up-left and up-right, or congruent and incongruent with visual motion, respectively.

Experiment 1

The PSEs, representing response biases (Figure S3C), were submitted to a two-way repeated measure ANOVA on the Sound (absent, stationary, left, and right) and SD level (five levels). Neither the main effects of Sound ($F(3,15) = 4.43$, $p = .076$, $\eta_p^2 = .47$) nor SD level ($F(1.21,6.07) = 0.94$, $p = .390$, $\eta_p^2 = .16$) was significant, and their interaction was not significant ($F(12,60) = 1.03$, $p = .438$, $\eta_p^2 = .17$).

The reciprocals of βs, representing the discrimination threshold of visual motion direction (Figure S3D), were submitted to a two-way repeated measure ANOVA on the factors of Sound (absent, stationary, congruent, and incongruent) and SD level (five levels). The main effect of SD level was significant ($F(1.97,9.87) = 53.97$, $p < .001$, $\eta_p^2 = .92$). *Post-hoc* pair-wise t-tests with Bonferroni correction demonstrated that thresholds were significantly higher at the 32° and 16° SD levels than their smaller SD levels, respectively (ps < .025). The main effect of Sound was significant ($F(3,15) = 6.70$, $p = .004$, $\eta_p^2 = .57$), and *post-hoc* pair-wise t-tests with Bonferroni correction indicate that thresholds were significantly higher in the incongruent and stationary conditions than in the congruent condition (ps < .027). However, the interaction between Sound and SD level was not significant ($F(12,60) = 1.34$, $p = .220$, $\eta_p^2 = .21$).

Finally, thresholds at each SD level across the four sound conditions were fitted with the EN model. The estimated internal noise and sampling efficiency were separately submitted to a one-way ANOVA on the factor of Sound (absent, stationary, congruent, and incongruent). Results showed that the internal noise was not significant ($F(3,15) = 0.27$, $p = .850$, $\eta_p^2 = .05$). However, the sampling efficiency was significant ($F(3,15) = 4.41$, $p = .021$, $\eta_p^2 = .47$), though *post-hoc* pair-wise t-tests with Bonferroni correction demonstrated no significant differences between any pairs of sound conditions (*p*s > .136). Taken together, even though the results of thresholds demonstrated an overall reduction in the congruent than in the incongruent and stationary conditions, no significant increase in sampling efficiency in the congruent condition was observed, similar to the results reported in the main text.

Experiment 2

The PSEs (Figure S3E) were submitted to a two-way repeated measure ANOVA on the factors of Sound (absent, stationary, up-left, and up-right) and SD level (five levels). Neither the main effect of Sound ($F(3,33) = 1.20$, $p = .32$, $\eta_p^2 = .10$) nor that of SD level ($F(1.97,21.68) = 1.97$, $p = .164$, $\eta_p^2 = .15$) was significant. Their interactions was marginally significant ($F(3.37,37.04) = 2.70$, $p = .054$, $\eta_p^2 = .20$).

The reciprocals of βs (Figure S3F) were submitted to a two-way repeated measure ANOVA on the factors of Sound (absent, stationary, congruent, and incongruent) and SD level (five levels). The main effect of SD level was significant ($F(1.38,15.16) = 82.20$, $p < .001$, $\eta_p^2 = .88$). *Post-hoc* pair-wise t-tests with Bonferroni correction demonstrated that the thresholds were significantly higher at the 32°, 16°, 8° and 4° SD levels compared to their respective smaller SD levels (*p*s < .001). However, the main effect of Sound was not significant ($F(3,33) = 0.19$, $p = .91$, $\eta_p^2 = .02$), nor was the interaction between Sound and SD level ($F(12,132) = 0.74$, $p = .71$, $\eta_p^2 = .06$).

Thresholds at each SD level across sound conditions were fitted with the EN model. The estimated internal noise and sampling efficiency were each analyzed with a one-way ANOVA on the factor of Sound (absent, stationary, congruent, and incongruent). Neither the internal noise ($F(3,33) = 0.63$, $p = .60$, $\eta_p^2 = .05$) nor the sampling efficiency ($F(3,33) = 0.37$, $p = .78$, $\eta_p^2 = .03$) was significant.

Reference

Prins, N., & Kingdom, F. A. A. (2009). *Palamedes: Matlab routines for analyzing psychophysical data.* http://www.palamedestoolbox.org

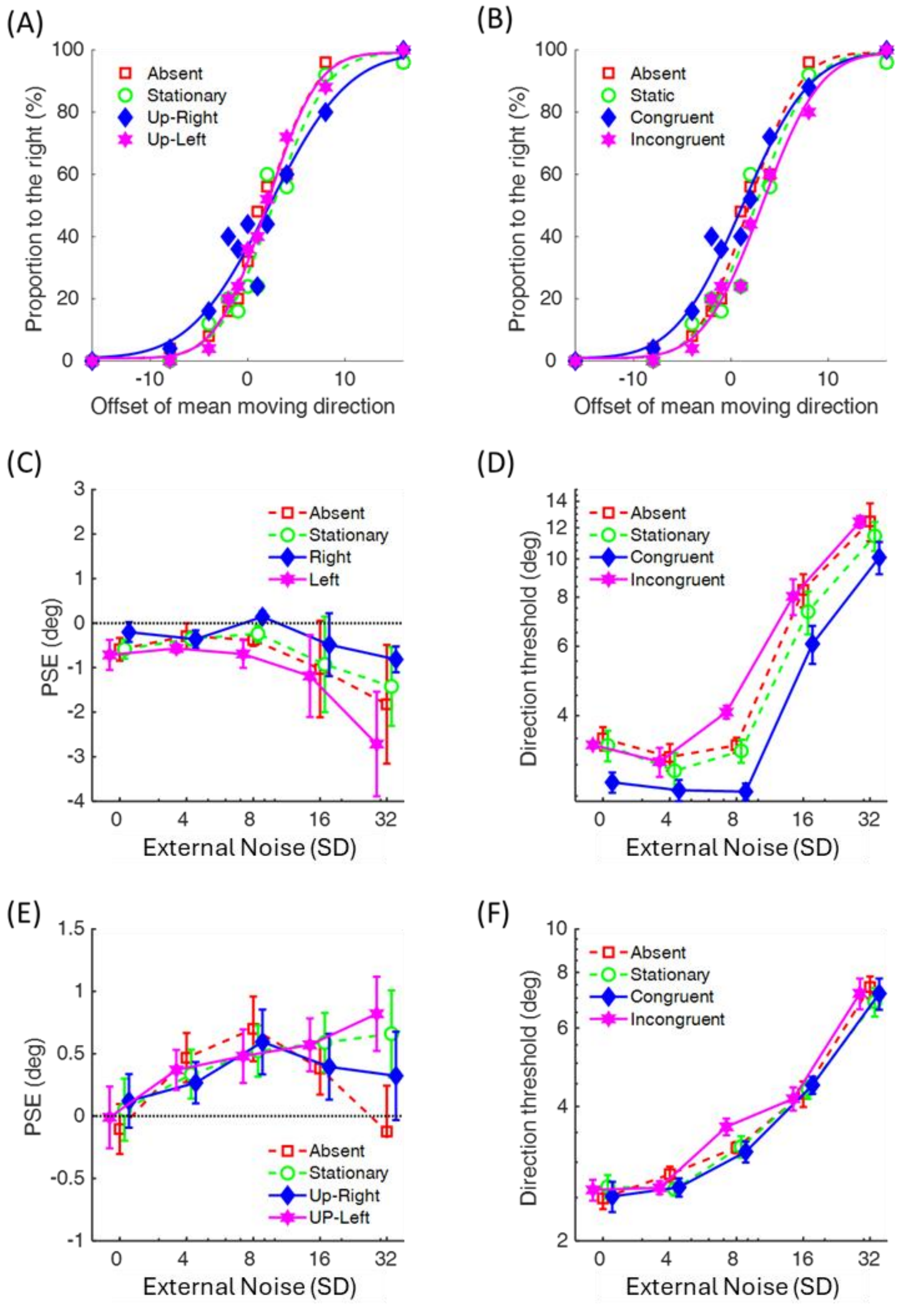

Figure S3. Data were reanalyzed using conventional methods, with the psychometric function defined as the proportion of perceived rightward motion relative to the actual left-to-right motion direction. The left column presented the results based on the direction of auditory motion, while the right column demonstrated the results based on the congruency between auditory and visual motions. (A) and (B) illustrate examples of psychometric functions for one participant in Experiment 2. (C) demonstrates the mean point of subjective equality (PSE) across six participants as a function of external noise (i.e., SD) levels in Experiment 1. (D) presents the mean Threshold vs. Noise (TvN) functions for Experiment 1 based on audiovisual congruency. (E) shows the mean PSE across 12 participants as a function of external noise (SD) levels in Experiment 2. (F) displays the mean TvN functions for Experiment 2, also based on audiovisual congruency.
